# Playing-Dumb Behavior of Trainers During Online Streaming and Trainee’s Burnout Behavior: Mediating Role of Psychological Disengagement

**DOI:** 10.3389/fpsyg.2021.819458

**Published:** 2022-02-10

**Authors:** Qing Xie, Shidong Li, Haider A. Malik, Supat Chupradit, Priyanut W. Chupradit, Abdul Qadus

**Affiliations:** ^1^School of Business and Economics, University Putra Malaysia, Seri Kembangan, Malaysia; ^2^School of Economics and Management, Anshun University, Anshun, China; ^3^Jeonju University, Jeonju, South Korea; ^4^Jinzhong University, Jinzhong, China; ^5^FAST School of Management, Islamabad, Pakistan; ^6^Department of Occupational Therapy, Faculty of Associated Medical Sciences, Chiang Mai University, Chiang Mai, Thailand; ^7^Educational Psychology and Guidance, Department of Educational Foundations and Development, Faculty of Education, Chiang Mai University, Chiang Mai, Thailand; ^8^Institute of Management Sciences (IMSciences), Peshawar, Pakistan

**Keywords:** playing dumb, psychological disengagement, burnout, trainees, trainers

## Abstract

A trainer’s behavior is a crucial factor, and it can affect the cognitive engagement of trainees in parts of training and development programs; thus, playing-dumb behavior by a trainer can cause lower attachment and less interested trainees during courses. This study was planned to investigate the impact of trainers’ playing-dumb behavior on trainees’ burnout behavior under the mediating role of psychological disengagement in online broadcasting. This study followed a convenience sampling technique under a cross-sectional research design, and data are collected from 371 trainees through a questionnaire. This study follows structural equation modeling to model the path relationship among study constructs. Results of this study indicate the presence of a relationship between trainers’ dumb behavior and its impact on trainee psychological disengagement, which leads to burnout. Findings of this study contribute to the inclusive body of knowledge pertaining to playing-dumb behavior and trainees’ burnout during the course.

## Introduction

Although there are available a plethora of literature that indicates knowledge hiding has been investigated at large in the past, the focus of this investigation remained on horizontal knowledge hiding (Connelly et al., 2012). Researchers have largely ignored how supervisors tend to hide knowledge from their subordinates. There are many possible reasons as to why supervisors might hide knowledge from their supervisees. A recent investigation by Butt and Ahmad (2020) compelled that supervisors/trainers usually possess some sort of special and unique knowledge that makes them significant and powerful in their circuits, and thus, to maintain their superior position and gain power, they might hide knowledge from their supervisee. Additionally, the desire to dominate to hold a prolonged career or even being afraid to be replaced by a competent supervisee or trainee can also generate a sense of completion or fear among the trainer, and he or she might hide knowledge from the trainee. Such a state of affairs can raise severe concern for modern-day organizations to manage the phenomena of knowledge hiding. Past studies document the phenomenon of knowledge hiding from many perspectives, such as from personality (Anand and Jain, 2014). In this line, other researchers, such as Demirkasimoglu (2016), explore knowledge hiding in the context of an extraversion personality trait and report that extroversion is positively correlated with knowledge hiding, whereas neuroticism was found in negative correlation with the “playing-dumb” knowledge-hiding behavior in academia. Other group-based or organizational dynamics have an impact on knowledge-hiding behaviors. Moreover, conflicts at the workplace can result in the shape of knowledge hiding.

Previous researchers largely address the knowledge management process in the shape of knowledge sharing, yet the literature on knowledge hiding from the perspective of our trainer’s knowledge-hiding behavior is scarce (Connelly et al., 2012). Additionally, previous researchers attempt to explore knowledge hiding from the perspective of personality psychology, ownership creativity, and leadership styles (Peng, 2013; Černe et al., 2014; Tang et al., 2015; Demirkasimoglu, 2016), yet there is no literature on the trainees’ reaction in response to the trainer’s knowledge-hiding behavior.

Although the literature indicates several negative consequences in response to knowledge hiding from individual to organizational levels, the severity and sensitivity of trainers’ knowledge-hiding behavior are still missing in past studies. Such hiding behavior can “trickle down” from trainers to trainees or supervisees, and they can also show knowledge-hiding behaviors (Mawritz et al., 2012). This can be more hazardous as compared with other types of knowledge hiding, such as horizontal knowledge hiding. Past studies indicate that each type of knowledge-hiding behavior has the potential to show a different effect on the various outcomes, so exploring the impact of each dimension would provide important insights (Ghasemaghaei and Turel, 2021).

**Figure 1:**
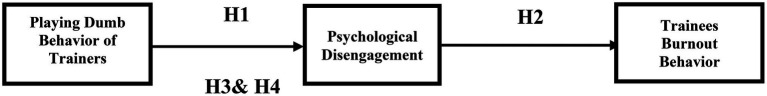
Conceptual model.

Past literature mainly focuses on knowledge-sharing processes (Gagné, 2009), thus, investigating the outcomes of knowledge hiding from the perspective of supervisors can enlighten the dark side of knowledge hiding to mitigate its poisonous outcomes. Thus, it would be imperative to investigate the consequences of supervisor’s knowledge-hiding behavior, particularly in the context of training. It would provide a more practical nuance to assess the trainer’s and trainee’s behaviors to improve the individual and organizational performance; thus, the first aim of this study is to investigate the impact of the trainer’s knowledge-hiding behavior and the trainee’s reaction. Moreover, literature indicates that playing-dumb behavior brings immediate psychological strain among the affected (Venz and Nesher Shoshan, 2021). Owing to this, the present study tends to address the shortcomings of the existing literature by exploring the impact of a trainer’s knowledge hiding on a trainee’s burnout behavior. Thus, this study makes several contributions to the existing body of knowledge. First, this study is the first, according to the authors’ knowledge, to have made an attempt to investigate trainers’ knowledge-hiding behavior, and second, this study anticipates the playing-dumb dimension of knowledge-hiding behavior of trainers during training sessions. Thus, this string also aims to extend the existing body of knowledge pertaining to counterproductive behaviors during training sessions. Third, this study also increases the understanding related to burnout, and finally, this study tests the relationship of playing-dumb behavior of trainers during online streaming and trainees’ burnout behavior through the mediating role of psychological disengagement, which is unique (Figure 1).

## Literature Review

### Knowledge Hiding

According to some scholars, knowledge hiding is also known as knowledge concealment. For instance, Lin and Wang (2012) describe knowledge withholding as hiding a piece of information that is significant and profitable to other people. On the other hand, Duffy et al. (2002) indicate that knowledge withholding is a type of social undermining in the workplace. By keeping the required information and creating belittling remarks about the target, a worker may be involved in vigorous or submissive sabotaging behaviors, including silent treatment. According to Connelly et al. (2012), intentional and active attempts are involved in knowledge hiding but “practices of neglecting to share knowledge accidentally and mishap or numbness” are not part of it. Knowledge hiding has different types, and every type results in different organizational behavior among employees, such as destroying organizational creativity, innovation, and performance. When people try to hide knowledge intentionally, they react in the same way when an employee requests knowledge from them (Connelly and Kevin Kelloway, 2003). On hiding knowledge, the knowledge keeper gets the selfish treatment that is shooting yourself in the foot. It means that interpersonal relationships among the employees may be demolished by mutual distrust (Černe et al., 2014). Knowledge hiding may also destroy the organizational performance by damaging the mutual understanding among the employees, creation of new thoughts, and implementation of policies (Peng, 2013). Knowledge hiding may also affect the employee’s creativeness (Černe et al., 2014).

Moreover, knowledge hiding includes various dimensions, which are labeled as (i) evasive hiding, (ii) playing dumb, and (iii) rationalized hiding (Connelly et al., 2012). In the first dimension of knowledge, that is, evasive hiding, individuals tend to hide knowledge from others by giving them false information or making false promises to share the requested knowledge. However, in the dimension of playing-dumb knowledge hiding, individuals tend to respond to knowledge requests by portraying that they do not have the requested knowledge. In rationalized hiding, individuals tend to provide excuses for failing to provide the knowledge requests.

Knowledge hiding usually occurs in the knowledge management system, in which an information request is made to a general audience or to make information to others (e.g., resources, reference materials, or documents) without any cause to do so (Wasko and Faraj, 2000; Babcock et al., 2004). The requested knowledge could be explicit or tacit in a way that it refers to skills and ideas that are not easily codified or that have been codified and can be explained, respectively (Nonaka and Takeuchi, 1995; Von Krogh et al., 2001). Another important factor of knowledge hiding is that the knowledge hider considers the knowledge as a limited resource, and he or she thinks that if he or she could share the knowledge, it could be lost.

In every situation, the knowledge hider does not hide knowledge intentionally; sometimes they hide knowledge to protect themselves or not to harm the feelings of others. There are various forms of knowledge hiding. One of them is evasive hiding in which the knowledge hider deceives the person and provides the wrong or misleading information to the requestor. The next form is playing dumb in which a person shows that he or she is unconscious of the requested knowledge. In this form, the knowledge hider also deceives the requestor. The third type of knowledge hiding is rationalized hiding, which is described as the hider explaining the reason for which he or she is not sharing the knowledge, and mostly, they blame a third party for not sharing the knowledge (Connelly et al., 2012).

### Psychological Disengagement

Based on social cognitive theory, it can be drawn safely that disengaged employees tend to deactivate themselves by compromising their self-regulatory mechanisms, which further can indulge them to be involved in a wide range of negative behaviors. In case someone is good at following the internal and external standards, he or she might deplete this resource, failing to cope with the negative behavior of the trainer, and he or she may experience stress in this regard (COR). The trainer’s knowledge-hiding behavior might be the reaction to unfair treatment within the organizational circuits. Owing to unfair and injustice treatment, employees tend to be morally disengaged, and they show a negative propensity to help others (Paciello et al., 2013). This concept is also proved in other disciplines, such as sports science, in which it has been tested that morally disengaged players tend to decrease their prosocial behavior (Kavussanu and Boardley, 2009). A simple decrease in prosocial behavior is linked with increased antisocial behaviors (Hodge and Gucciardi, 2015). Knowledge hiding is considered a positive act as influenced by prosocial motivation and is considered as less harmful owing to the nature of hiding. In this regard, Connelly et al. (2012) state that knowledge hiding based on rationalized hiding is not harmful because it can be due to prosocial motivation, and it can increase the bond strength between the knowledge seeker and knowledge hider (Connelly and Zweig, 2015). However, researchers and practitioners place more emphasis on the need to understand and explore the more negative consequences of knowledge hiding. Moreover, some researchers have also recommended that exploring knowledge-hiding factors/predictors of knowledge hiding can provide important insights. In the same vein, some researchers also stress the need to investigate what organizations can do to decrease knowledge hiding (Xiao and Cooke, 2019).

### Trainee Burnout

Job stress occurs when a person feels a significant gap between the imposed job demands and the person’s knowledge, skills, abilities, or another type of resource required to meet the demands and perceives himself as unable to cope with the situation. It is the situation in which the person analyses the job demands or stressors faced and the available resources and fails to find enough resources to cope with these demands. Having sufficient resources may help to cope with the demands in a more efficient way. For example, personal resources, such as self-efficacy, self-esteem, optimism, hope, resilience, knowledge, and experience, give you some sense of control over the environment and social resources that provide you with social support (both emotional or practical) and facilitation from people around you. Individuals with greater resources can respond to stressors more effectively. On the other hand, resource deficiency makes them ineffective and less responsive to external demands. Such circumstances make them more vulnerable to stress and may lead them to severe physical and mental deterioration.

People respond to the same situations in different ways. Stress levels vary from person to person even if they are exposed to the same situations and challenges. How much a person is threatened by stress does not depend on the actual stressful event or situation faced. It depends on how the person interprets and perceives this situation. People respond to these external events as they perceive them; that is why their response and stress levels vary from each other under the same circumstances. Although stress factors usually arise from the outside, how you respond to them comes from inside. The relationship between an individual and his or her environment can either be stressful or not. According to the European Agency for safety and health at work, job stress is not only a major reason for absenteeism, turnover, and low productivity, but it is also the second most important factor in health-related problems in employees, including high blood pressure and heart attacks. In Europe, approximately 30% of workers suffer from stress, 23% have fatigue, 11.2% have irritability, 8% have anxiety, and 9% are facing sleeping problems (Milczarek et al., 2009). According to the Health and Safety Executive United Kingdom, “Stress accounted for 35% of all work-related ill health cases and 43% of all working days lost due to ill health.” Work stress is not a hot topic to only a few countries, but it is a global issue. In 1993, a United Nations report tagged job stress as “the 20th century disease,” and after a few years, the WHO labeled work stress a “worldwide epidemic.”

Burnout is positively associated with employees’ poor health outcomes, so it is not only a moral obligation, but also in the best interests of organizations to prevent and manage job stress among their employees. They must implement systematic interventions for the prevention of stress-related issues and ensure the mental health and safety of their employees by providing a healthy environment (Kasperczyk, 2010).

Job stress is considered a bone of contention in an organizational environment due to its toxic consequences. Many organizations conduct stress management programs that include consultancy, job timing flexibility, social support, providing assistance, job redesign, recreational activities, sports, nutrition, exercises, relationship management (Kantor et al., 1997), and play therapy help to reduce employees’ stress (Nel and Spies, 2007).

Two basic approaches to stress at work are distinguished in the literature: transactional (Lazarus and Folkman, 1984) and interactionism (Karasek, 1979). The theory of conservation of resources of Hobfoll (1989) explains the mechanism by which individuals explain stress at the workplace when they meet with resource drainage. Stress is defined as “a reaction to the environment that leads to (a) the threat of loss of resources, (b) the net loss of resources, or (c) a lack of resource gain following a significant investment of resources” (Hobfoll, 1989).

According to the American Psychological Association (2014), 78% of American adults are suffering from stress, and 69% of the stress is associated with work. Forty-eight percent of employees in the United Kingdom are close to burnout due to prolonged stress. A survey on working conditions in Europe is conducted every 5 years by European foundations for the improvement of living and working conditions; it reports that stress levels at work have been increasing for the last few years due to job insecurity; long working hours; pressure to increase speed; hard deadlines; lack of job control; poor work–life balance; bullying; harassment; conflicts with supervisor, coworkers, or clients; discrimination; or poor communication within the organization. Prolonged stress can destroy the psychological and physical health of workers. The report states, “Work-related stress is one of the biggest health and safety challenges that we face in Europe” (Milczarek et al., 2009; Irastorza et al., 2016).

Some researchers in the past note that stress is not always destructive. It depends on whether the stress response is generated from positive emotions or negative; thus, two types of stress are found: “eustress” and “distress.” Eustress is referred to as stress with positive implications, and distress is related to negative events in one’s life (Selye, 1974). The performance–stress relationship is an inverted U-shaped curve that shows performance increases along with the increase in stress, but this increase in performance ceases at a certain point and starts declining beyond that point. At a moderate level of stress, employees are highly motivated for problem-solving and coping with situations. This approach leads them to higher performance.

### Relationship of Playing-Dumb Behavior of Trainer and Psychological Disengagement

Behind the trainer’s knowledge-hiding behavior, there may be some reasons, such as silence, which may be termed as counterproductive work behavior in the shape of a negative reaction. This negative reaction as counterproductive work behavior can be intentional holding of knowledge, information, or even ideas from the trainees (Milliken et al., 2003; Morrison, 2014). Previous researchers identify silence as dangerous for individuals and organizations because it has the potency to hamper individual performance (Bolton et al., 2012), and it can result in poor performance of the organization too (Morrison, 2014).

The premise of adaptive cost theory explains the outcomes related to a trainer’s knowledge-hiding behavior. A trainer’s knowledge hiding might bring disengagement in trainees as a result of knowledge-hiding behavior because, when a trainer seeks to withhold and hide knowledge from his or her trainees, it assures the trainees that their trainer is not showing interpersonal help in the shape of knowledge hiding (Kelloway and Barling, 2000). This can be the result of increasing pressure or a sense of competition, which might force trainers to hide knowledge, for instance, when a trainer or supervisor perceives a threat from the environment or perceives that he or she might be replaced with a new one tends to cope with these demands/stressors by showing less or decreasing in interpersonal helping.

The engagement theory presented by Kahn (1990) predicts that individuals tend to be engaged in their in-role behaviors under the right circumstances. Engagement is measured as a motivational concept and is expressed as “the instantaneous service and appearance of a person’s chosen self in task comportments that endorse linkings to exertion and to others, personal presence (physical, cognitive, and emotional), and active, full role concerts” (Kahn, 1990). Although contrary to this, disengagement could be realized as a deficiency and exertion of energy, emotion, and thought formally defined as “the instantaneous amputation and defense of a person’s favorite self in comportments that promote a lack of connections, physical, cognitive, and emotional absence and passive, inadequate role recitals.” Thus, trainers’ knowledge-hiding behavior might trigger trainees to withdraw from positive behaviors, and consequently, they can become prey to disengagement. Thus, on the basis of the above argument, the following can be hypothesized:

*H1:* Playing-dumb behavior of a trainer is associated with psychological disengagement.

### Relationship of Trainee Psychological Disengagement and Trainee Burnout

Presently, these days, work stress is becoming greater in the surroundings of work. Mursali et al. (2009) characterize that stress at slog (business-related stretch or aptitude and vocation anxiety) as the enthusiastic reactions and destructive physical condition that happen when someone’s job prerequisites do not contend with the resources, capacities, or necessities possessed by that person. Nowadays job stress is growing widely in the settings of work, and this has grabbed attention on work stress and its unmistakable consequences on the return of several features of organizations. Mursali et al. (2009) define trauma and strain at work (work-related or job stress) as the emotional responses that happen when the job requirements never compete for resources, capacities, or necessities of the employees. Research suggests that increasing stress at the job motivates members of a staff to show negative behavior. Employment stress at the job has turned into a usually pessimistic growing number that has, as a consequence of overtask, job instability. Prolonged stress is found to be the cause of various diseases, such as heart attacks, stroke, hypertension, kidney disease, etc. Initially it was perceived only as a disease and threat to physical and mental health.

Lazarus and Folkman (1984) postulate that trauma occurs “when a separate perceives that the claims of an external ailment and complaints are elsewhere his or her perceived capacity to endure and survive with them.” In other words, people get stressed when they feel a lack of control over the situation or find themselves unable to fulfill the demands imposed on them. They further explain that, if a person succeeds in coping with the stressor, stress remains harmless and may provide a feeling of achievement or fulfillment. In case of failure to cope with the situation, stress becomes out of control and appears as a destructive thing. Thus, on the basis of this argument, the following can be safely assumed:

*H2:* Psychological disengagement is associated with trainee burnout behavior.

### Mediating Role of Psychological Disengagement Between the Relationship of Playing-Dumb Behavior of Trainer and Trainee Burnout

The premise of social exchange theory, developed by Blau (1964), can also be used in this scenario to enlighten the affiliation between trainer knowledge hiding, trainee psychological disengagement, and trainee burnout. Under the economic exchange premise of this theory, it can be safely predicted that trainees receiving a lack of sustenance in the shape of knowledge hiding might develop a state of negative emotions (Molm, 1994). Moreover, according to the premise of this theory, behaviors at the workplace are stimulated by “the longing to exploit positive understandings and minimalize undesirable and damaging involvements through social alliances” (Weiss and Stevens, 1993). Simply, when trainees distinguish and observe that they are not being treated fairly and their trainer is hiding knowledge from them and they consider that there is less organizational support for them, they can experience burnout as a reaction to this (Serenko and Bontis, 2016), and a negative relationship leads to negative behaviors.

The phenomenon of knowledge hiding is triggered or persuaded by the self-focused intentions of the knowledge hiders. On the other hand, knowledge sharing is an outcome of the prosocial intentions of the individuals (Pan et al., 2018). Owing to this, it can be undertaken that knowledge hiding is different from other similar concepts/constructs, such as knowledge hoarding (Webster et al., 2008). In knowledge hoarding, knowledge is kept hidden intentionally, whereas knowledge hiding occurs when someone requests knowledge. It is also confirmed by past researchers (Connelly et al., 2012) that these two constructs are different from each other.

Notably, knowledge hiding is not the contradictory of concept of knowledge sharing. For instance, employees may choose not to share knowledge due to the absence of knowledge or the unawareness of the chance to share (Zhao et al., 2019).

The term “eustress” is considered good stress, whereas on the contrary, “distress” is labeled as bad stress. When the word “stress” is used alone, it usually refers to distress. Stress definitions and how it occurs vary. Lazarus and Folkman (1984) expand their research on stress and state that stress occurs in an individual when “demands exceed the personal and social resources the individual can mobilize.” They explain stress as a relationship between an individual and the environment in which external demands are evaluated or perceived as exceeding their resources and threatening their wellbeing. They put stressors that arose from the environment on one side of the balance and personal characteristics and resources on the other side. Their research manifests that a person is more likely to be stressed whenever the equilibrium deteriorates between the external demands and the individual’s resources.

It is axiomatic that effective knowledge management presents several benefits to both the organization and its members in contemporary work contexts (Collins and Smith, 2006; Su and Daspit, 2021). Although a variety of efforts have been made in knowledge management to facilitate effective knowledge transfer within organizations, knowledge hiding is still pervasive (Connelly et al., 2019). Knowledge hiding refers to an intentional attempt to conceal or withhold knowledge that others have requested and encompasses three dimensions, namely, evasive hiding (promising to share but not sharing), playing dumb (pretending not to know), and rationalized hiding (giving a reason for not sharing; Connelly et al., 2012). Knowledge hiding captures an intentional or deliberate act of withholding knowledge (Connelly et al., 2012).

High stress is perceived as a greater risk or threat, so it leads employees toward emotional defensive coping mechanisms rather than problem-solving coping, which is positively associated with performance. Eustress is a certain level of stress that may provide an increased level of energy and can be beneficial. It is the optimal level of stress at which the nervous system is highly active, adaptive, and raises the performance levels. It does not cause any damage or harm. When the construct distress is beyond the optimal point, it decreases one’s performance and causes exhaustion, frustration, and burnout. It poses a threat to the mental and physical health of the individuals. Thus, on the basis of these arguments, it can be assumed that employees in response to a trainer’s knowledge-hiding behavior will psychology disengage and ultimately develop burnout, so the following is hypothesized:

*H3:* Playing-dumb behavior of a trainer is associated with trainee burnout.

*H4:* Psychological disengagement mediates the relationship of playing-dumb behavior of trainers and trainee burnout behavior.

## Research Methods

This study aims to investigate the playing-dumb behavior of trainers during online streaming and trainees’ burnout behavior through the mediating role of psychological disengagement. This study used a deductive strategy to evaluate the hypotheses as in a deductive approach hypotheses are provided based on prior literature and then assessed using various analysis approaches to get results. This study used primary data sources, and data were collected *via* a convenience sampling technique from the respondents because this technique provides flexibility to collect the data from individuals who are easily available and willing to engage using convenience random sampling. Initially, the HR department of various organizations were approached and ensured that their organization provided online training to their employees. After ensuring it from concerned HR managers, contact details of respective employees were requested, and then, those employees/trainees were approached for data collection. They were briefed about the study purpose, and their participation was sought. After obtaining informed consent, they were asked to provide their feedback and their behavior in online streaming. The sample size is determined in this manuscript by the total number of items; hence, 371 responses are being used for analysis. A few simple demographic questions were asked, such as age, gender, and education, to ascertain the authenticity of the data. These questions were asked keeping in view the general characteristics of the respondents.

### Instrument Development

In this research, we developed a measuring scale for all these constructs using previous indications. The responses were rated using a 5-point Likert scale ranging from 1 (strongly disagree) to 5 (strongly agree). We investigated the reliability and validity of all constructs through Smart-PLS. In total, 12 items were used to measure all three constructs: playing-dumb behavior of trainers, psychological disengagement, and trainee burnout behavior. Playing dumb was measured through indicators used in prior studies (Bari et al., 2019) and developed by Connelly et al. (2012), which consist of four items. All the measurement items of psychological disagreement were adapted from Caudroit et al. (2010) and six items in this regard were chosen to measure psychological disagreement. Finally, the scale for trainee burnout behavior was measured through the scale proposed by the authors (Escartín et al., 2017) having two items.

### Data Analysis Technique

This study used the Smart-Partial Least Square Structural Equation Modeling identified as PLS-SEM (Sarstedt et al., 2017; Hair et al., 2017a). Data analysis in this regard is based on assessment of the (i) measurement model and (ii) structural model. The measurement assessment explains the measurements of all variables in the model, and the structural model assessment identifies the relationship among variables in the model. The measurement model estimation involves the reliability of indicators and constructs in the research model. In addition, it comprises both sorts of construct validities: discriminant and convergent. There are certain estimates that measure the reliability and validity of the constructs and indicators. Among them, factor loadings estimate the indicator reliability (FD), construct reliability (CR), and Cronbach alpha (α) both estimate construct reliability. Moreover, the average variance extracted (AVE) is considered for convergent validity, Fornell and Larcker criterion, and HTMT ratio for discriminant validity. These measures have threshold points where FD, CR, and α should be greater or equal to 0.70 (Hair et al., 2011; Sarstedt et al., 2017); however, the AVE value should be equal to or greater than 0.50 (Sarstedt et al., 2017). In the case of the Fornell and Larcker criterion, the square root of all diagonal values should be higher than the off-diagonal values. Values of HTMT should be less than 0.85 (Ab Hamid et al., 2017).

## Analysis and Findings

The demographic summary is illustrated in Table 1 (gender, age, and education). The overall summary demonstrates that males and females are almost equally considered to generalize the outcomes of results as 46.24% are male respondents and 54.03% are female. Most of the trainee respondents 69% have an average age between 20 to 30 years, and they are highly qualified doctorate 25.54%, master’s 29.84%, and bachelor’s 24.19, and 86.82% of trainees were involved in trainings during online streaming.

**Table 1:** Demographic details of respondents.

Demographic details	Respondents	%
**Gender**		
Male	170	46.24
Female	201	54.03
**Age**		
20 and fewer years	50	13.44
21 to 25	61	16.40
26 to 30	99	26.61
31 to 35	51	13.71
36 to 40	39	10.48
41 to 45	35	9.41
46 to 50	36	9.95
**Education**		
Bachelor and lower	90	24.19
Master	110	29.84
Doctorate	95	25.54
Diploma and others	76	20.43
**Are you involve/involved during online streaming?**		
Yes	322	86.82
No	49	13.17
Total	*n* = 371	

### Reliability Analysis and Measurement Model Estimation

**Figure 2:**
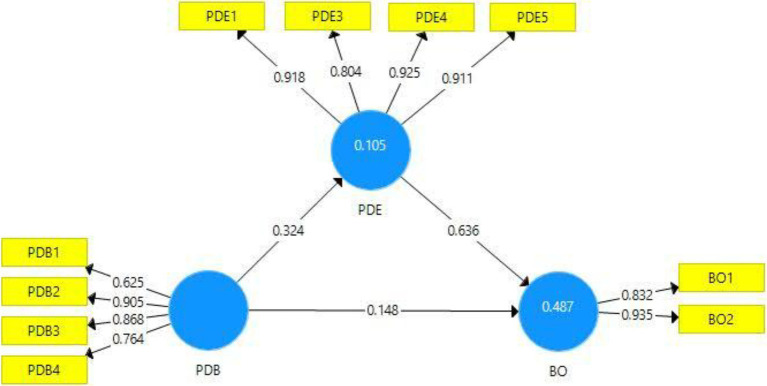
Measurement model.

Factor loadings are also used to assess the dependability of indicators or items within constructs (Isaac et al., 2019). The coefficients of 0.70 or above show significant factor loadings (Hair et al., 2017a) although the factor loading ≥0.5 is accepted (Hair et al., 2010). As a result, Figure 2 shows that every factor loading is over the criteria of 0.708 (except PDB1); thus, every item meets the requirement of factor loadings, and items are reliable to measure the unique variable. Hence, the indicator reliability is maintained. Moreover, two items from the construct psychological disengagement were dropped due to poor outer loadings.

Table 2 delineates the reliability of the constructs and their items. The reliability trustworthiness, convergent, and discriminant validity of conceptions and measures are used to evaluate measurement models. For determining the reliability of the construct, Cronbach alpha (α) coefficients were evaluated, and α was contemplated to be consistent if its value is >0.7 (Hair et al., 2017b), and the α range in this measurement model was 0.735—0.913, which all were reliable. According to Gefen et al. (2011) and Hair et al. (2017b), values of composite reliability should be >0.7, and all values of CR in this phenomenon were above 0.7, which all were valid for assessment. As a result, the construct reliability criteria have been fulfilled and satisfied, and the Cronbach alpha and CR coefficients have been calculated and coefficients are well above the threshold point. In terms of construct reliability, it may be claimed that all of the constructs have good reliability.

**Table 2:** Reliability analysis and measurement model estimates.

Construct	Cronbach’s alpha	rho_A	Composite reliability	Average Variance Extracted (AVE)
BO	0.735	0.839	0.878	0.783
PDB	0.805	0.859	0.873	0.637
PDE	0.913	0.924	0.939	0.793

BO = Burnout behavior, PDE = Psychological disengagement, PDB = Playing-dumb behavior by trainer.

Furthermore, the AVE is used to quantify the constructs’ convergent validity; it truly evaluates the positive connection between the items of homologous constructs (Isaac et al., 2019). The values of AVE fell in between 0.637 and 0.793 and likewise should be greater than 0.5 (Hair et al., 2010); hence, values have fulfilled the threshold.

Finally, the discriminant validity is tested using the Fornell and Larcker criterion and the HTMT heterotrait–monotrait ratio of correlation (Ab Hamid et al., 2017). The coefficients in Table 3 show that the discriminant validity was adequate. As stated, the larger diagonal values obtained by the Fornell and Larcker ratio than their below values suggest substantial association across constructs, the diagonal values in Table 3 are greater than underneath values. Likewise, the HTMT values are below the ratio of 0.85 as stated by Lia et al. (2020), whereas values near 0.90 indicate issues in data. As a result, all values in Table 4 are within the range and less than 0.85, indicating that the HTMT results provide sufficient proof for discriminant validity.

**Table 3:** Fornell and Larcker.

Construct	BO	PDB	PDE
BO	**0.885**		
PDB	0.353	**0.798**	
PDE	0.684	0.324	**0.891**

BO = Burnout behavior, PDE = Psychological disengagement, PDB = Playing-dumb behavior by trainer. Bold values are square root of AVE of respective construct.

**Table 4:** HTMT ratio.

Construct	BO	PDB	PDE
BO	–		
PDB	0.425	–	
PDE	0.795	0.371	–

### Structural Model Assessment

**Figure 3:**
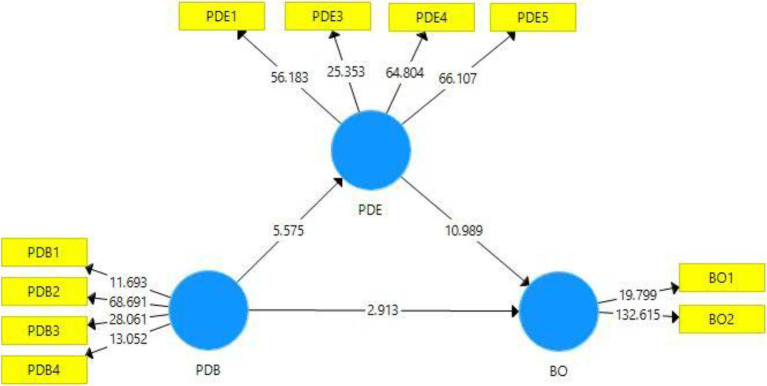
Smart-PLS bootstrapping outcomes.

The structural model assessment (Figure 3) involved the valuation of Beta (β), respective value of *p*, and *t*-figures through the bootstrap procedure with 5,000 resamples in Smart-PLS 3.3.3 (Hair et al., 2017b). Values of *p* and *t*-statistics are used to analyze the statistical significance.

Table 5 illustrates the hypotheses status on the basis of empirical results. The first hypothesis of this study pertains to the relationship of playing-dumb behavior of the trainers and psychological disengagement of the trainees (H1). This hypothesis is accepted on the basis of *t* and *p* statistics (Table 5), and it can be concluded that individuals, when they experience playing-dumb knowledge-hiding behavior of trainers, they tend to psychologically disengage. Similarly, in the case of hypotheses 2, which is regarding the relationship of psychological disengagement and trainees’ burnout behavior, the results are statistically accepted, and this hypothesis is supported (on the basis of *t* and values of *p*), thus confirming that trainees experience burnout when they are psychologically disengagement. For the third hypothesis of this study (H3), that is, playing-dumb behavior of the trainers and burnout behavior of the trainees, the results are statistically accepted on the basis of *t* and values of *p*, and it can be concluded that individuals experiencing trainers’ playing-dumb behavior develop burnout (H3). As concerns the fourth hypothesis of this study, it was tested on the basis of variance account for approach (VAF), which is obtained by dividing indirect effect by total effect. Hence, VAF in this case has been observed as 58%, which indicates a situation of partial mediation, thus confirming H4.

**Table 5:** Hypotheses testing.

	Hypothesis	Coefficient (Beta)	*SD*	*t*	*p*	Status
H1	PDB > PDE	0.325	0.058	5.575	0.000	Supported
H2	PDE > BO	0.637	0.058	10.989	0.000	Supported
H3	PDB > BO	0.153	0.051	2.913	0.004	Supported
	Mediation analysis		Indirect effect	Total effect	VAF	Status
H4	PDB > PDE > BO		0.207	0.36	58%	Supported

BO = Burnout behavior, PDE = Psychological disengagement, PDB = Playing-dumb behavior by trainer.

## Discussion and Implications

This study explores the association between the playing-dumb behavior of trainers and trainees’ burnout behavior during online streaming through the mediating role of psychological disengagement. Our findings also imply that playing-dumb behavior of trainers significantly leads to trainee burnout because humans learn from their experiences showcasing mental representations of tasks and activities; if the trainers behaves dumbly to trainees, then they automatically show their burnout behavior (Simon and Hayes, 1976). Usually, high rates of burnout behavior are associated with increased dumb behavior of trainers during online streaming. Because, when people share depression, they may shield their self-esteem by undervaluing or tumbling the emotional significance to their self-concept (Crocker and Major, 1989). It is also highlighted that sometimes another reason behind burnout behavior is psychological changes (Perseius et al., 2007). Trainee burnout and depression were associated with avoidance and decreased engagement in the values-based behavior of trainers. Sometimes trainers may not meet the requirements of trainees when the organizations have not hired suitable trainers who are unable to motivate trainees in online streaming.

In previous research, there are several predictors of burnout behavior in different industry and country settings (Giorgi et al., 2016; Escartín et al., 2017; Cotel et al., 2021; De la Fuente-Solana et al., 2021; Guidetti et al., 2021; Nishimura et al., 2021; Serrão et al., 2021). However, the current research uniquely investigates the psychological disagreement and playing-dumb behavior by trainers, which consequently causes increased burnout among trainees. Moreover, burnout has been allied with constituent abuse, hopeless ideation, and occupational unhappiness. Healthy pecuniary performers can lead to and may smooth specialized and personal freedoms to mitigate burnout-associated trainee behavior. Moreover, in such a situation when trainees show burnout behavior due to dumb behavior of trainers, then spiritual disengagement plays an important role in the relationship of trainers and trainees. As psychological disengagement apparatuses resemble an intellectual evacuation from academic achievement so that it is no longer considered a primary source of self-esteem (Tougas and Beaton, 2008; Martinot et al., 2020). Therefore, the examination of psychological underpinnings of academic disengagement involves the exploration of its links with self-esteem, especially in such situations (Orth and Robins, 2014; Martinot et al., 2020). Hence, our findings are correlated with previous research that psychological disengagement significantly mediates the relationship among trainees playing dumb and trainees’ burnout behavior. So, the organizations should focus on selecting trainers in online streaming because the behavior of trainers has a significant impact on the trainees’ behavior; if the trainers behave in a dumb manner, it will exaggerate the burnout behavior of trainees. Moreover, the organizations can lessen this negative behavior by introducing the psychological disengagement construct as it significantly mediates the relationship among trainers and trainees in online streaming.

## Conclusion

To conclude this study, the dumb behavior of trainers has a crucial impact on the burnout behavior of trainees in online streaming. Interestingly, psychological disengagement plays an important role in the relationship of trainers and trainees. Additionally, this study has considered the mediating role of psychological disengagement in playing-dumb behavior of trainers and trainee burnout behavior during online streaming. It can be concluded safely that playing-dumb behavior of trainers has a hazardous impact on the behavior of trainees, which leads to burnout. Moreover, the mediating role of psychological disengagement was considered and significantly mediates the relationship between the dumb behavior of trainers and the burnout behavior of trainees.

This study also offers ample prospects for future researchers to copy this study model in other contexts and settings. Besides this, the existing model can be extended by investigating the dimensional influence of psychological disengagement on trainers’ and trainees’ behavior in online streaming. Moreover, in future studies, other dimensions of knowledge-hiding behavior can also be considered as a predictor of psychological disengagement. Future researchers can also consider other possible mediators, such as psychological withdrawal and emotional exhaustion. Similarly, future studies can also consider other outcome variables to document the impact of playing-dumb knowledge-hiding behavior.

## Data Availability Statement

The original contributions presented in the study are included in the article/supplementary material, and further inquiries can be directed to the corresponding author.

## Ethics Statement

All subjects gave their informed consent for inclusion before they participated in the study. The study was conducted in accordance with the Declaration of Helsinki, and the protocol was approved by the Jeonju University, China.

## Author Contributions

QX and SL conceived and designed the concept. HM collected the data, provided technical support, and wrote the paper. SC and PC have read and agreed to the published version of the manuscript. QX finalize the review. AQ made corrections in conclusion and finalize the review. All the author contributed for this manuscript.

## Conflict of Interest

The authors declare that the research was conducted in the absence of any commercial or financial relationships that could be construed as a potential conflict of interest.

## Publisher’s Note

All claims expressed in this article are solely those of the authors and do not necessarily represent those of their affiliated organizations, or those of the publisher, the editors and the reviewers. Any product that may be evaluated in this article, or claim that may be made by its manufacturer, is not guaranteed or endorsed by the publisher.
